# Localization of phosphorylated ErbB1-4 and heregulin in colorectal cancer

**DOI:** 10.1186/1471-2407-14-863

**Published:** 2014-11-22

**Authors:** Keigo Mitsui, Masaoki Yonezawa, Atsushi Tatsuguchi, Seiichi Shinji, Katya Gudis, Shu Tanaka, Shunji Fujimori, Choitsu Sakamoto

**Affiliations:** Division of Gastroenterology, Department of Internal Medicine, Nippon Medical School, 1-1-5 Sendagi, Bunkyo-ku, Tokyo, 113-8603 Japan; Department of Analytic Human Pathology, Nippon Medical School, Tokyo, Japan; Department of Surgery, Nippon Medical School, Tokyo, Japan

**Keywords:** Colorectal cancer, Heregulin, EGFR, ErbB2, ErbB3, ErbB4, Phosphorylation, Nuclear localization

## Abstract

**Background:**

The ErbB family consists of four proteins including (EGFR)/ErbB1, ErbB2, ErbB3, and ErbB4, and plays a crucial role in the promotion of multiple tumorigenic processes. In addition to the traditional pathways of EGFR signaling, EGFR translocates to the nucleus and acts as a transcription factor in the proliferation of cancer cells. Heregulin is known as both an ErbB3 and an ErbB4 ligand. This study aimed to investigate the expression of heregulin and its relevant EGFR family members as well as their phosphorylated forms in human colorectal cancer (CRC) tissues and to determine the relationship between their expression and clinicopathological factors including patient prognosis.

**Methods:**

We analyzed the effects of exogenous heregulin on ErbB2, ErbB3 and ErbB4 phosphorylation in Caco-2, DLD-1, and HCT 116 colon cancer cell lines by western blot analysis. We examined 155 surgical resections from colorectomy patients. Cellular localization of ErbB1-4, their phosphorylated forms and heregulin protein was analyzed in CRC surgical resections by immunohistochemical analysis. Immunohistochemical results were compared with clinicopathological factors and patient prognosis.

**Results:**

Phosphorylated ErbB2 (pErbB2) and phosphorylated ErbB3 (pErbB3) were detected in both nuclear and cytosolic fractions of Caco-2 and DLD-1 cells stimulated by exogenous heregulin. Whereas, phosphorylated ErbB4 (pErbB4) was detected only in cytosolic fractions of HCT 116 cells stimulated by exogenous heregulin. Phosphorylated EGFR (pEGFR) immunoreactivity was observed in the cytoplasm and nuclei of cancer cells, whereas the pattern of EGFR staining was membranous and cytoplasmic. Subcellular localization of pErbB2, cytoplasmic, membranous, or nuclear, varied among cases. pErbB3 immunoreactivity was exclusively observed in the nuclei of cancer cells. pErbB4 immunoreactivity was observed in the cell membrane of cancer cells. Statistically, heregulin immunoreactivity correlated with pErbB2 and pErbB4 expression. In multivariate analysis for disease free survival, lymph node status, pErbB3 and pErbB4 expression retained independent prognostic significance. In multivariate analysis for overall survival, lymph node status, pEGFR and pErbB4 retained independent prognostic significance.

**Conclusions:**

ErbB2 and ErbB3 phosphorylated by heregulin localized in the nucleus of CRC cells. Phosphorylated ErbB1-4 and heregulin contribute to poorer patient prognosis in CRC. This heregulin-ErbB family member autocrine loop may be a candidate for targeted treatment of CRC.

**Electronic supplementary material:**

The online version of this article (doi:10.1186/1471-2407-14-863) contains supplementary material, which is available to authorized users.

## Background

The epidermal growth factor receptor (EGFR) family consists of four receptors; ErbB1/HER1/EGFR, ErbB2/HER2, ErbB3/HER3, and ErbB4/HER4, and belongs to the superfamily of type I receptor tyrosine kinases. The EGFR and all other family members contain an extracellular ligand-binding domain (except in the case of ErbB2), a transmembrane region and a cytoplasmic domain with kinase activity [1, 2]. Activation of EGFR receptors by the binding of the cognate ligands induces formation of homo- and hetero-dimers between different members of the EGFR family, and subsequent phosphorylation of specific sites in the cytoplasmic tail and recruitment of protein adaptors results in activation of multiple downstream signaling pathways. ErbB receptors have been studied for their use as potential prognostic markers and therapeutic targets [3, 4].

Colorectal cancer (CRC) remains a significant cause of morbidity and mortality throughout the world [5, 6]. The low molecular weight tyrosine kinase inhibitors and the monoclonal antibodies against EGFR are in clinical development and have shown clinical benefit in metastatic CRC [7, 8]. EGFR and ErbB2, two of four members of the EGFR tyrosine kinase family, have been widely studied on CRC [4]. In contrast, the remaining members of the family, ErbB3 and ErbB4, have been largely ignored by clinical studies targeting CRC [4]. It has been reported that intestine-specific ErbB3 ablation resulted in almost complete absence of intestinal tumors in the *Apc*^Min^ mouse model of colon cancer and that ErbB3-ErbB4 heterodimers contribute to colon cancer survival [9]. Nevertheless, the relationship between the expression of these proteins and clinicopathological factors, as evaluated by immunohistochemical techniques, has proven to be controversial in those studies [4].

In addition to the traditional pathways of EGFR signaling, several studies have reported that EGFR translocates to the nucleus and acts as a transcription factor in the proliferation of normal and cancer cells alike [10, 11]. For example, nuclear EGFR has been detected in a number of human cancers including breast, oropharyngeal, bladder, pancreatic, ovarian, lung and colorectal [12–14], and nuclear EGFR has been associated with acquired resistance to chemotherapy and poor prognosis [15–17]. On the other hand, there have only been a few studies concerning the clinical significance of nuclear or phosphorylated EGFR family members in human CRC.

The identities of the activating ligand and the heterodimer partner are the most important factors in determining which pathway is activated. The ErbB2-ErbB3 hetero-dimer is considered the most potent ErbB pair with respect to strength of interaction, ligand-induced tyrosine phosphorylation and downstream signaling, and functions as an oncogenic unit [18, 19]. Heregulin is a member of the EGF-family peptides and is known as both an ErbB3 and an ErbB4 ligand [20]. Interaction of heregulin with the dimers of its receptors, including ErbB3 and ErbB4, results in various biological functions. To date, most studies have shown that heregulin exhibits a growth-stimulatory effect in a variety of cancer cells, including breast cancer cells and colorectal cancer cells [21, 22]. Previously we have shown that heregulin may contribute to angiogenesis through the phosphorylation of ErbB3 in colon cancer cell lines [23]. However, there have been scant reports regarding the relationship between heregulin expression and clinicopathological factors including patient prognosis, and the relationship between each member of the EGFR family and heregulin in human CRC [24].

This study aimed to investigate the expression of heregulin and its relevant EGFR family members, EGFR, ErbB2, ErbB3 and ErbB4, and their phosphorylated forms, in human CRC tissues and to determine the relationship between their expression and clinicopathological factors and patient prognosis.

## Methods

### Materials

Recombinant human heregulin-β1 EGF domain (rHRG) was purchased from R&D System Inc. (Minneapolis, MN.). The list of primary antibodies used in this study is shown in Additional file 1: Table S1. Of these, anti-EGFR antibody (clone EGFR.25; Leica biosystems/Novocastra) is raised to the cytoplasmic domain of the EGFR molecule. Anti-ErbB2 antibody (Dako) is raised to the cytoplasmic domain of the ErbB2 molecule. Anti-ErbB3 antibody (abcam) is a synthetic peptide corresponding to an internal sequence of ErbB3. Anti-ErbB4 antibody (Thermo scientific) immunogen is a C-terminus of human ErbB4.

### Cell culture

The human colorectal cancer cell lines Caco-2, DLD-1 and HCT 116 cells were routinely grown according to the supplier’s instructions, supplemented with 10% fetal calf serum.

### Immunoprecipitation and Western blot analysis

Colon cancer cells were starved in serum-free medium overnight before rHRG treatment. Then, cells were incubated with or without 100 ng/mL rHRG for 1 h at 37°C. Nuclear extracts were obtained according to Schreiber et al. [25]. Typically, 1 × 10^6^ cells from cultured cell lines were washed with 10 mL cold PBS and pelleted by centrifugation at 1500 *g* for 5 min. The pellet was resuspended in 1 mL PBS, and pelleted again by spinning for 3 min in a microfuge. PBS was removed and the cell pellet resuspended in 400 μL cold buffer A (10 mM HEPES pH7.9; 10 mM KCL; 0.1 mM EDTA; 0.1 mM EGTA; 1 mM DTT; 0.5 mM PMSF; 1 mM Vanadate) on ice for 15 min, after which 25 μL of a 10% solution of NP-40 was added and the tube vortexed for 10 sec. The tube was then centrifuged at 500 *g* for 3 min and the non-nuclear fraction obtained from the supernatant. The nuclear pellet was resuspended in 200 μL ice-cold buffer B (20 mM HEPES pH 7.9; 0.4 M NaCl; 1 mM EDTA; 1 mM EGTA; 1 mM DTT; 1 mM PMSF; 1 mM Vanadate) at 4°C for 15 min. The tube was then centrifuged at 15000 *rpm* for 15 min at 4°C and the nuclear fraction obtained from the supernatant. The nuclear fractions were normalized by total protein amount (1 mg) before immunoprecipitation. One mg protein samples were incubated with 3 μg of anti-phosphotyrosine antibody (PY-20; Santa-Cruz, CA) immobilized onto protein G-Sepharose for 4 h at 4°C. Immunoprecipitates were washed thrice with washing buffer (50 mM HEPES (pH 7.6), 150 mM NaCl, 0.1% Triton X-100) and boiled 5 min in SDS sample buffer. The samples were separated by 10% SDS-polyacrylamide gel electrophoresis and transferred to a PVDF membrane. The transferred proteins were probed with specific antibodies against ErbB2 (C-18; Upstate, NY), ErbB3 (C-17; Santa-Cruz, CA) and pErbB4 (Tyr1162; Cell Applications inc., San Diego, CA) for 1 h at 25°C. After washing, protein signals were detected with horseradish peroxidase-conjugated antibody against the appropriate IgG using enhanced chemiluminescence detection.

### Patients and tissue samples

We obtained 155 colon and rectum adenocarcinoma tissue samples from archives of the Department of Pathology at Nippon Medical School Hospital for immunohistochemical analysis of heregulin, EGFR, ErbB2, ErbB3, ErbB4, pEGFR, pErbB2, pErbB3 and pErbB4 protein expression. Patients included 90 men and 65 women ranging in age from 44 to 91 years (average age, 66.1 years; median, 66.0 years). We excluded patients who had undergone chemotherapy or radiation. Patients were traced via hospital and pathology records from 1996 to 2006. Disease free survival (DFS) was defined as the interval from the date of the first surgery until relapse, the appearance of a second primary cancer, or death, whichever occurred first. At the time of analysis, 47 patients had died, and 108 still survived. The median follow-up time for the whole series was 42 months (mean, 46 months; range, 3 to 111 months) and the median survival 62 months (mean, 56 months; range, 3 to 111 months). All subjects gave informed consent, and the project was approved by the Ethics Committee of Nippon Medical School. All staging criteria were defined according to the International Union Against Cancer TNM classifications.

### Immunohistochemical analysis

Specimens were fixed in 10% formalin, embedded in paraffin wax, cut into 4 μm sections, and immersed in 0.3% H_2_O_2_–methanol for 30 min to block endogenous peroxidase activity. Sections were then microwaved in 0.01 mol/l citrate phosphate buffer (pH 6.0) or EDTA (pH9.0) for antigen retrieval and incubated with 10% normal horse or goat serum for 10 min at 37°C to block nonspecific immunoglobulin (IgG) binding. Thereafter, sections were incubated for 18 h at 4°C with anti-heregulin, anti-EGFR, anti-pEGFR, anti-ErbB2, anti-pErbB2, anti-ErbB3, anti-pErbB3, anti-ErbB4, or anti-pErbB4 antibodies. They were then treated with their respective biotinylated antibodies; namely, anti-mouse IgM, anti-mouse IgG, or anti-rabbit IgG (1:200) for 1 h at 25°C, followed by treatment with avidin-biotin peroxidase complex for 1 h at 25°C. The reaction products were developed by immersing sections in 3,3′-diaminobenzidine tetrahydrochloride solution containing 0.03% H_2_O_2_. Nuclei were counterstained with Mayer’s hematoxylin.

### Evaluation of immunohistochemical staining

Each case was evaluated blindly by two independent observers (K.M. and A.T.). Any disagreement was resolved using a multi-headed microscope (kw, Heregulin = 85%; kw, EGFR = 80%; kw, ErbB2 = 82%; kw, ErbB3 = 90%; kw, ErbB4 = 88%; kw, pEGFR = 82%; kw, pErbB2 = 78%; kw, pErbB3 = 90%; kw, pErbB4 = 82%). Cases showing heregulin or ErbB3 immunostaining were scored using the following scoring system adopted by Rajkumar, resulting from the product of the score for the fraction of positive cells (range, 0 to 4 [0, <10% positively stained cells; 1, 10% to 25%; 2, 26% to 50%; 3, 51% to 75%; and 4, >75%]) and the score for staining intensity (range, 0 to 3). Slices with scores of 8 or higher were classified as positive and slices with scores lower than 8 as negative. EGFR, ErbB2, ErbB4 or pErbB4 staining was scored semi-quantitatively according to the following scoring system approved by the US Food and Drug Administration [26]: 0, no immunostaining or membrane staining in <10% of the tumor cells; 1+, incomplete membrane staining of >10% of tumor cells; 2+, weak-to-moderately complete membrane staining of >10% of tumor cells; 3+, moderate-to-strongly complete membrane staining of >10% of tumor cells. Scores of 0 or 1+ indicated tumor negative, and scores of 2+ and 3+ were regarded as positive expression of EGFR, ErbB2, ErbB4, and pErbB4.

Subcellular localization of pErbB2 varied among cases; cytoplasmic, membranous, or nuclear. Cases showing pErbB2 immunoreactivity in >10% percent of cancer cells were regarded as positive irrespective of subcellular localization. Subcellular localization of pErbB3 was exclusively in the nucleus. There was no variation in the intensity of nuclear staining and only cases with more than 10% of cells showing nuclear pEGFR or pErbB3 were considered positive.

### Statistical analysis

Immunostaining results for each protein were compared with clinicopathological factors including age, gender, location, vessel invasion, node metastasis, depth of invasion, and stage, using the chi-square test or Fisher’s exact test as appropriate. The association among each protein immunostaining was also assessed by the chi-square test or Fisher’s exact test as appropriate. The distribution of disease free survival was estimated by Kaplan-Meier methodology, and the log-rank test was used to test for significant differences in disease free survival. A Cox proportional hazard model was used to assess the effect of tumor variables on overall survival. In multivariate analysis, variables with P < 0.05 in the univariate analysis were included. A P value of <0.05 was considered significant.

## Results

### Cancer cell lines

**Figure 1:**
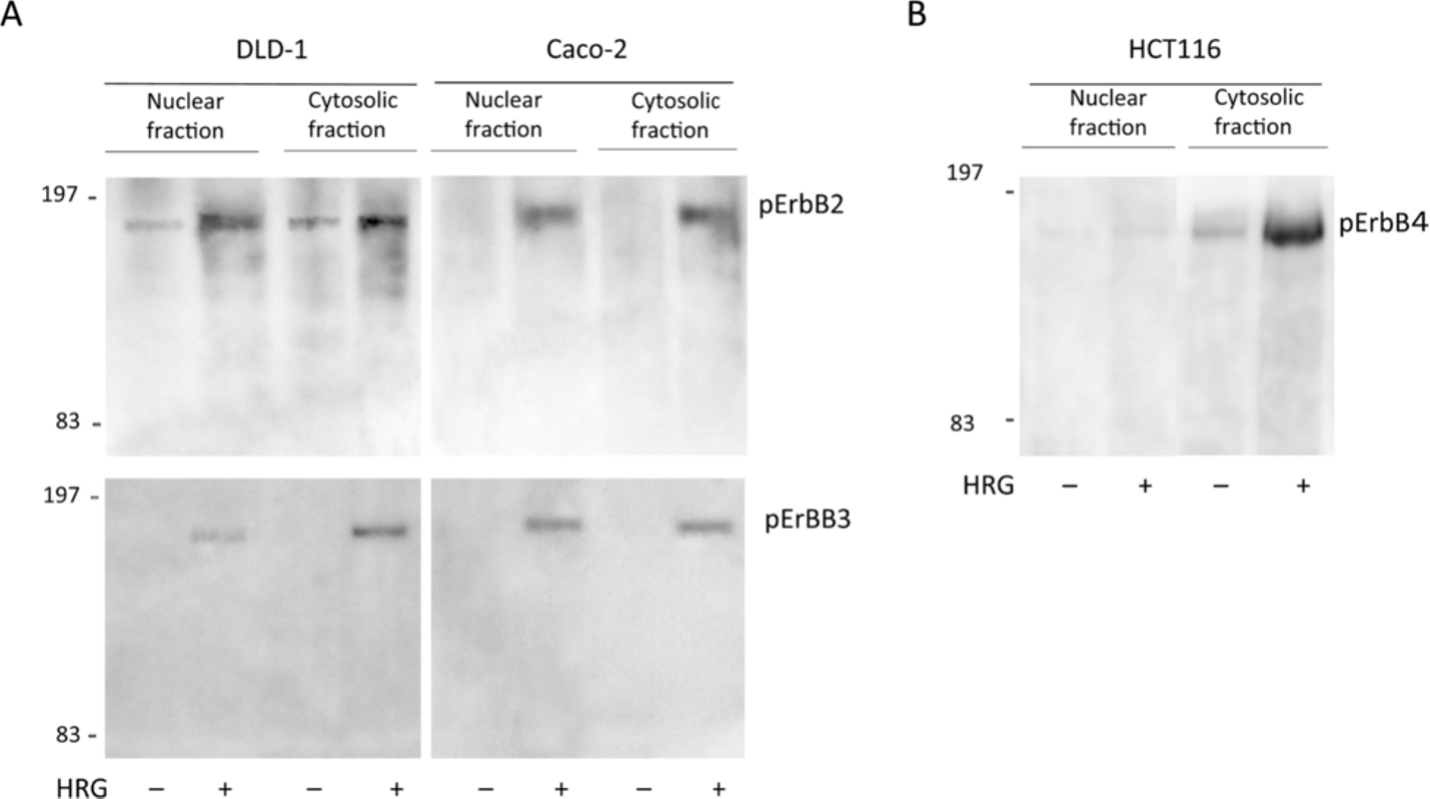
**The effects of exogenous heregulin on tyrosine phosphorylation of ErbB2, ErbB3 and ErbB4 in both the cytosolic and nuclear fractions of colon cancer cell lines.** After heregulin treatment, the cells were lysed and immunoprecipitated with an antibody against phosphotyrosine (PY-20). Immunoprecipitates were analyzed by Western blotting with anti-phosphorylated ErbB receptor antibodies. **A**, pErbB2 and pErbB3 were detected in both the nuclear and cytosolic fractions of both DLD-1 and Caco-2 cells after heregulin stimulation. **B**, ErbB4 phosphorylation increased with exogenous heregulin stimulation in only the cytosolic fractions of HCT 116 cells.

Previously, we examined the effects of rHRG on tyrosine phosphorylation of ErbB proteins in cytosolic fractions of colon cancer cell lines [23]. In this study we examined the effects of rHRG on tyrosine phosphorylation of the ErbB2, ErbB3, and ErbB4 proteins in nuclear fractions of colon cancer cell lines.After immunoprecipitation with an antibody against phosphotyrosine (PY-20), western blot analyses revealed that both the DLD-1 and Caco-2 cell lines express a protein with a molecular weight of approximately 180 kD reacting against antibodies to the ErbB2 (C-18) and ErbB3 (C-17) proteins (Figure 1A). Although ErbB2 phosphorylation was observed under basal conditions, exogenous rHRG further stimulated ErbB2 in both the nuclear and cytosolic fractions of DLD-1 cells. On the other hand, pErbB2 was detected in the nuclear fraction of Caco-2 cell only after rHRG treatment. The same was true for the expression of pErbB3 in DLD-1 and Caco-2 cells, where pErbB3 was detected in both the nuclear and cytosolic fractions following cellular exposure to rHRG. These results suggest that exogenous rHRG stimulated ErbB2 and ErbB3 phosphorylation in the nuclear fractions of both DLD-1 and Caco-2 cells. The limitation of this study revolves around the fact that calculation of the relative amounts of pErbB2 and pErbB3 in the cytoplasm and nuclear fractions in cancer cell lines is not warranted given the data. Prior to immunoprecipitation, the nuclear and cytoplasmic fractions of cancer cell lines were normalized by total protein amount (1 mg) rather than by the number of cell equivalents of each fraction.

ErbB4 was not detected in any fractions in DLD-1 or Caco-2 cells (data not shown) [23]. Therefore, our present study used the HCT 116 cell line to detect ErbB4 according to Xu et al. [27]. After immunoprecipitation by PY-20, a western blot analysis using an antibody against ErbB4 (Tyr1162) revealed a band of approximately 180 kD in the cytosolic fraction of HCT 116 cells in the absence of rHRG stimulation. ErbB4 phosphorylation increased with rHRG stimulation in the cytosolic fractions of HCT 116 cells. ErbB4 was nearly absent in the nuclear fractions of HCT 116 cell regardless of ligand stimulation (Figure 1B). This suggests that although heregulin stimulates ErbB4 phosphorylation on the cell membrane, pErbB4 fails to translocate into the nucleus.

### Heregulin, ErbB1-4, and pErbB1-4 localization in colorectal cancer

**Figure 2:**
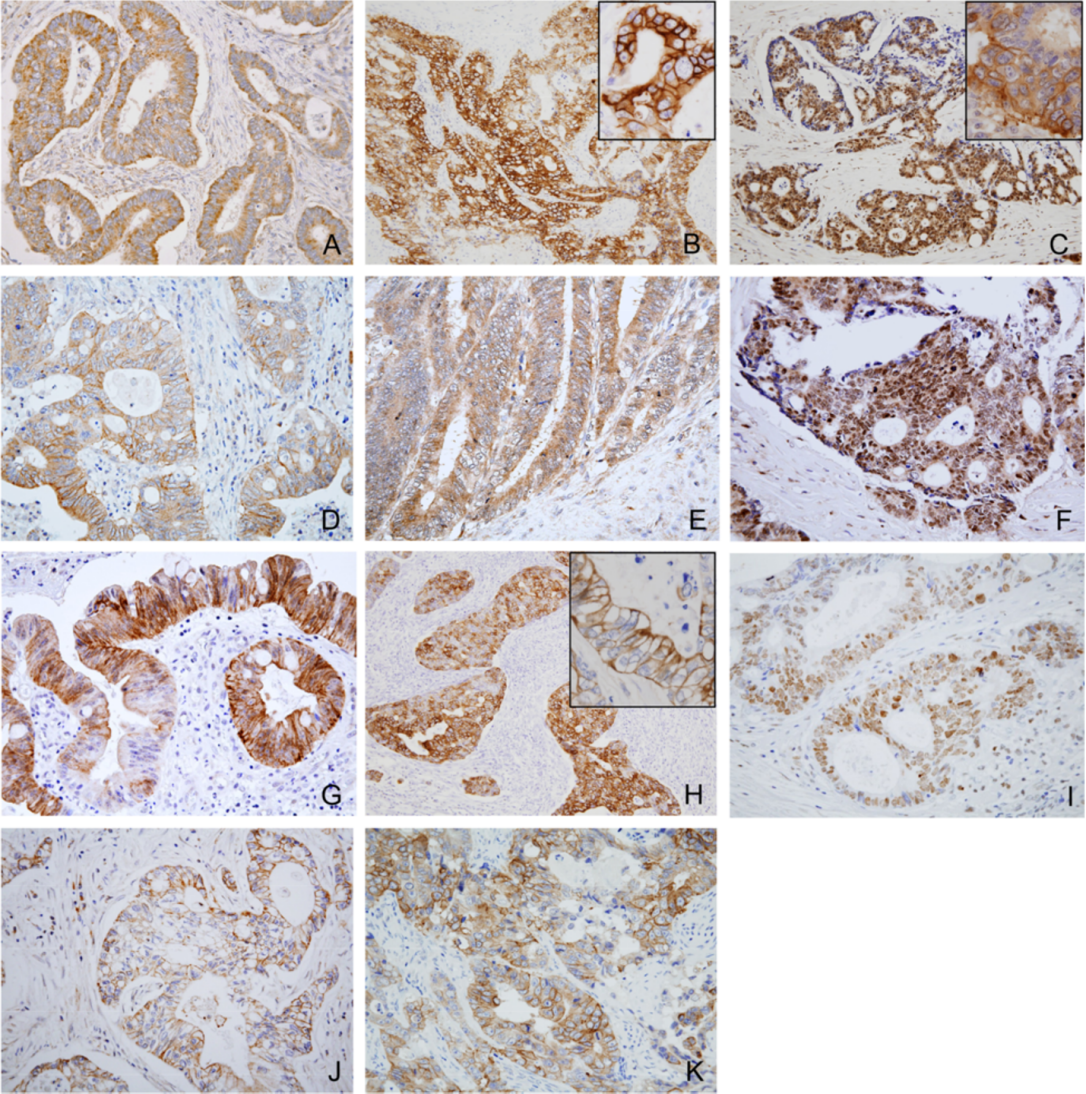
**Immunohistochemical localization of heregulin (A), EGFR (B), pEGFR (C), ErbB2 (D), pErbB2 (E-G), ErbB3 (H), pErbB3 (I), ErbB4 (J), and pErbB4 (K) in colorectal adenocarcinoma.** **A**, Heregulin was stained in the cytoplasm of adenocarcinoma cells. 40x **B**, EGFR was stained in the cell membrane and cytoplasm of adenocarcinoma cells. 20x, Insert; EGFR in the cell membrane. 60x **C**, pEGFR was stained in the nucleus of adenocarcinoma cells. 20x, Insert; pEGFR in the cell membrane and cytoplasm. 60x **D**, ErbB2 was stained in the cell membrane of adenocarcinoma cells. 40x **E**, pErbB2 was stained in the cytoplasm of adenocarcinoma cells. 40x **F**, pErbB2 was stained in the cell membrane of adenocarcinoma cells. 40x **G**, pErbB2 was stained in the nucleus of adenocarcinoma cells. 40x **H**, ErbB3 was stained in the cell membrane and cytoplasm of adenocarcinoma cells. 20x, Insert; ErbB3 in the cell membrane. 60x **I**, pErbB3 was stained in the nucleus of adenocarcinoma cells. 40x **J**, ErbB4 was stained in the cell membrane of adenocarcinoma cells. 40x **K**, pErbB4 was stained in the cell membrane of adenocarcinoma cells. 40x.

We next examined localization of heregulin, EGFR, ErbB2, ErbB3, ErbB4, pEGFR, pErbB2, pErbB3 and pErbB4 proteins by immunohistochemical analysis.Heregulin immunoreactivity was predominantly observed in the cytoplasm of cancer cells in 72 cases (46%; 31% with score 8, 9% with score 9 and 6% with score 12) (Figure 2A). Heregulin expression was weak-to-absent in stromal cells, including fibroblasts, but was nearly absent in glandular epithelial cells of colorectal mucosa adjoining cancer tissue.EGFR immunoreactivity was observed in cancer cells in 79 cases (51%; 40% with score 2+ and 10% with score 3+). The predominant pattern of EGFR staining was membranous and cytoplasmic (Figure 2B). pEGFR immunoreactivity was observed in the cytoplasm and nuclei of cancer cells in 50 (32%) cases (Figure 2C). EGFR and pEGFR were nearly absent in glandular epithelial cells of colorectal mucosa adjoining cancer tissue.ErbB2 immunoreactivity was observed in cancer cells in 41 cases (26%; 16% with score 2+ and 10% with score 3+). The predominant pattern of ErbB2 staining was in the membrane (Figure 2D); a few tumors showed only cytoplasmic staining but these cases were considered negative according to the evaluation method followed. Subcellular localization of pErbB2 varied among cases; cytoplasmic (Figure 2E), membranous (Figure 2F), and/or nuclear (Figure 2G). When considering pErbB2 immunoreactivity as positive irrespective of subcellular localization, 30 (19%) cases were found to be positive for pErbB2, 21 (14%) cases were cytoplasmic, 1 (1%) case was membranous and 8 (5%) cases were nuclear. ErbB2 and pErbB2 were nearly absent in glandular epithelial cells of colorectal mucosa adjoining cancer tissue.ErbB3 immunoreactivity was predominantly observed in the cytoplasm and membrane of cancer cells in 64 (41%) cases (Figure 2H), whereas pErbB3 immunoreactivity was exclusively observed in the nuclei of cancer cells in 40 (26%) cases (Figure 2I). ErbB3 and pErbB3 were nearly absent in glandular epithelial cells of colorectal mucosa adjoining cancer tissue.ErbB4 and pErbB4 immunoreactivity was observed in cancer cells in 33 (21%) and 25 (16%) cases, respectively (Figure 2J,K). The predominant pattern of ErbB4 and pErbB4 staining was membranous with or without cytoplasmic expression. ErbB4 and pErbB4 were nearly absent in glandular epithelial cells of colorectal mucosa adjoining cancer tissue.

### Relationship between heregulin, ErbB1-4, pErbB1-4 and clinicopathological factors

We then explored the clinical significance of the protein expression of these receptors and heregulin in CRC. For tumors that were considered as positive irrespective of the subcellular localization of each protein, the relationship with clinicopathological factors was analyzed statistically in Table 1.

**Table 1:** **Relationship between heregulin, ErbB1-4, pErbB1-4 and clinicopathological factors**

		Heregulin		EGFR		pEGFR		ErbB2		pErbB2		ErbB3		pErbB3		ErbB4		pErbB4	
	No.	No. (percent)	P value	No. (percent)	P value	No. (percent)	P value	No. (percent)	P value	No. (percent)	P value	No. (percent)	P value	No. (percent)	P value	No. (percent)	P value	No. (percent)	P value
Age																			
<66	78	38 (49)	NS	46 (59)	NS	27 (35)	NS	14 (18)	0.018	12 (15)	NS	28 (36)	NS	19 (24)	NS	17 (22)	NS	9 (12)	NS
≥66	77	33 (43)		33 (43)		23 (30)		27 (35)		18 (23)		36 (47)		21 (27)		16 (21)		16 (21)	
Gender																			
Male	90	41 (46)	NS	45 (50)	NS	26 (29)	NS	27 (30)	NS	20 (22)	NS	41 (46)	NS	22 (24)	NS	17 (19)	NS	16 (18)	NS
Female	65	30 (46)		34 (52)		24 (37)		14 (22)		10 (15)		23 (35)		18 (28)		16 (25)		9 (4)	
Site																			
Colon	106	45 (42)	NS	52 (49)	NS	34 (32)	NS	29 (27)	NS	20 (19)	NS	40 (38)	NS	25 (24)	NS	23 (22)	NS	19 (18)	NS
Rectum	49	26 (53)		27 (55)		16 (33)		12 (24)		10 (20)		24 (49)		15 (31)		10 (20)		6 (12)	
Depth																			
pT1	12	4 (33)	0.03	4 (33)	0.004	0	0.003	1 (8)	NS	0	0.015	1 (8)	0.003	0	0.015	0	0.004	0	0.009
pT2	29	8 (28)		7 (24)		4 (14)		5 (17)		5 (17)		6 (21)		4 (14)		1 (3)		0	
pT3	105	52 (50)		62 (59)		42 (40)		30 (29)		20 (19)		52 (50)		35 (33)		31 (30)		24 (23)	
pT4	9	7 (78)		6 (67)		4 (44)		5 (56)		5 (56)		5 (56)		1 (11)		1 (11)		1 (11)	
Lymphatic invasion																			
Negative	31	9 (29)	0.044	11 (35)	NS	8 (26)	NS	7 (23)	NS	4 (13)	NS	12 (39)	NS	8 (26)	NS	3 (10)	NS	3 (10)	NS
Positive	124	62 (50)		68 (55)		42 (39)		34 (27)		26 (21)		52 (42)		32 (26)		30 (24)		22 (18)	
Vascular invasion																			
Negative	61	24 (39)	NS	25 (41)	0.05	12 (20)	0.008	15 (25)	NS	10 (16)	NS	19 (31)	0.046	8 (13)	0.005	10 (16)	NS	7 (11)	NS
Positive	94	47 (50)		54 (57)		38 (40)		26 (28)		20 (21)		45 (48)		32 (34)		23 (24)		18 (19)	
Lymph node status																			
pN0	85	37 (44)	NS	35 (41)	0.01	18 (21)	0.002	18 (21)	NS	11 (13)	0.04	34 (40)	NS	18 (21)	NS	18 (21)	NS	15 (18)	NS
pNx	70	34 (49)		44 (63)		32 (46)		23 (33)		19 (27)		30 (43)		22 (31)		15 (21)		10 (14)	
TNM stage																			
I	35	11 (31)	0.023	9 (26)	<0.001	3 (9)	<0.001	5 (14)	0.033	4 (11)	0.031	7 (20)	0.026	3 (8)	0.041	1 (3)	<0.001	0	0.005
II	43	21 (49)		20 (47)		11 (26)		9 (21)		4 (9)		21 (49)		11 (26)		13 (30)		11 (26)	
III	53	22 (42)		31 (58)		20 (38)		16 (31)		14 (26)		23 (43)		17 (32)		8 (15)		7 (13)	
IV	24	17 (71)		19 (79)		16 (67)		11 (46)		8 (33)		13 (54)		9 (38)		11 (46)		7 (29)	
Liver metastatis																			
Negative	133	55 (41)	0.001	62 (47)	0.01	35 (26)	<0.001	30 (23)	0.016	22 (17)	0.041	52 (39)	NS	32 (24)	NS	22 (17)	0.001	18 (14)	NS
Positive	22	16 (73)		17 (77)		15 (68)		11 (50)		8 (36)		12 (55)		8 (36)		11 (50)		7 (32)	
Recurrence																			
Negative	119	51 (43)	NS	55 (46)	0.037	29 (24)	<0.001	28 (24)	NS	19 (16)	NS	40 (34)	0.001	21 (18)	<0.001	18 (15)	0.002	12 (10)	0.001
Positive	36	20 (56)		24 (67)		21 (58)		13 (36)		11 (31)		24 (67)		19 (53)		15 (42)		13 (36)	

Positive rates for each heregulin, pEGFR, ErbB2, pErbB2, and ErbB3 immunostaining increased significantly with invasion depth. Positive rates for EGFR, pEGFR, ErbB2 and pErbB3 immunostaining increased significantly with advancing stage. Heregulin and, with the exception of ErbB3, EGFR family member immunoreactivities correlated with liver metastasis (the case of pErbB4 was borderline; P value = 0.057).

Each phosphorylated ErbB receptor immunoreactivity correlated with the corresponding ErbB receptor; namely, pEGFR correlated with EGFR (Additional file 2: Table S2). Since heregulin induces ErbB2 phosphorylation via both ErbB3 and ErbB4 phosphorylation, heregulin expression was predicted to correlate with the expression of all three phosphorylated forms, pErbB2, pErbB3 and pErbB4. However, although heregulin immunoreactivity correlated with pErbB2 and pErbB4, no correlation was found with pErbB3 expression. Instead, EGFR and pEGFR were found to correlate with pErbB2, pErbB3 and pErbB4. In addition, pErbB2 correlated with pErbB3 and pErbB4 (Additional file 2: Table S2). This stands to reason because pErbB2 can be induced via either ErbB3 or ErbB4 phosphorylation.

### Comparative survival analysis

Finally, we explored the relationship between clinicopathological factors and each protein expression, and patient prognosis of CRC.

Survival analysis was performed on 95 stage II-III patients for disease free survival; stage I patients were omitted since none of the stage I patients had died during the follow-up time. In univariate analysis using the Cox proportional hazards model for disease free survival, lymph node status, heregulin, pEGFR, pErbB2, ErbB3, pErbB3, ErbB4 and pErbB4 each had significant prognostic value (Table 2). However, in multivariate analysis performed by introducing all the above variables in the Cox proportional hazards model, lymph node status, pErbB3 and pErbB4 expression retained independent prognostic significance (Table 2).

**Table 2:** **Univariate and multivariate Cox proportional hazards analysis for disease-free survival (Stage ll-lll) (N = 95)**

Variables	Categories	Univariate analysis		Multivariate analysis	
		HR (95% CI)	***P***value	HR (95% CI)	***P***value
Depth (T factor)	T2 vs. T3, T4	1.40 (0.82-2.38)	NS		
Lymph node status	Positive vs. negative	2.55 (1.25-5.20)	0.01	3.46 (1.50-7.97)	0.004
Heregulin expression	Positive vs. negative	2.03 (1.04-3.94)	0.037	1.72 (0.83-3.55)	NS
EGFR expresion	Positive vs. negative	1.76 (0.88-3.51)	NS		
pEGFR expression	Positive vs. negative	4.08 (2.08-8.01)	<0.001	2.06 (0.94-4.55)	NS
ErbB2 expression	Positive vs. negative	1.83 (0.93-3.62)	NS		
pErbB2 expression	Positive vs. negative	2.90 (1.44-5.81)	0.003	1.05 (0.47-2.34)	NS
EbB3 expression	Positive vs. negative	2.24 (1.14-4.44)	0.02	1.31 (0.61-2.85)	NS
pErbB3 expression	Positive vs. negative	3.76 (1.95-7.28)	<0.001	2.60 (1.06-6.37)	0.036
ErbB4 expression	Positive vs. negative	2.27 (1.15-4.49)	0.018	0.69 (0.21-2.35)	NS
pErbB4 expression	Positive vs. negative	3.41 (1.25-5.20)	<0.001	5.24 (1.50-7.97)	0.01

**Figure 3:**
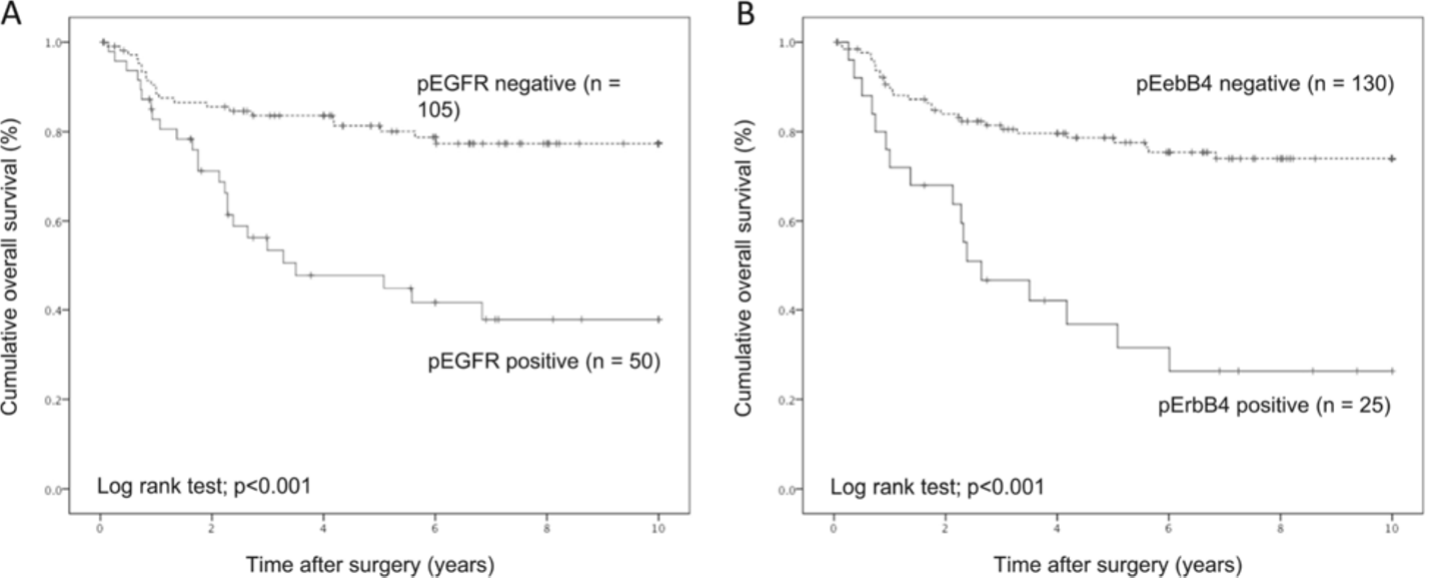
**Kaplan-Meier overall survival curve for pEGFR expression (A) and pErbB4 expression (B) in stage l-lV patients.**

Survival analysis was performed on 155 stage I-lV patients for overall survival. In univariate analysis using the Cox proportional hazards model for overall survival, lymph node status, heregulin, EGFR, pEGFR, ErbB2, pErbB2, ErbB3, pErbB3, ErbB4 and pErbB4 each had significant prognostic value (Table 3). In multivariate analysis performed by introducing all the above variables in the Cox proportional hazards model, lymph node status, pEGFR and pErbB4 retained independent prognostic significance (Table 3, Figure 3). Similar results were obtained for stage II-IV stage patients (data not shown).

**Table 3:** **Univariate and multivariate Cox proportional hazards analysis for overall survival (stage l-lV) (N = 155)**

Variables	Categories	Univariate analysis		Multivariate analysis	
		HR (95% CI)	***P***value	HR (95% CI)	***P***value
Depth (T factor)	T1, T2 vs. T3, T4	1.78 (0.95-3.53)	NS		
Lymph node status	Positive vs. negative	5.32 (2.75-10.32)	<0.001	6.27 (2.94-13.36)	<0.001
Heregulin expression	Positive vs. negative	2.22 (1.23-4.01)	0.008	1.81 (0.94-3.46)	NS
EGFR expression	Positive vs. negative	2.31 (1.25-4.27)	0.007	0.45 (0.16-1.27)	NS
pEGFR expression	Positive vs. negative	3.93 (2.19-7.05)	<0.001	2.85 (1.05-7.73)	0.04
ErbB2 expression	Positive vs. negative	1.95 (1.08-3.51)	0.027	0.59 (0.16-2.15)	NS
pErbB2 expression	Positive vs. negative	2.29 (1.23-4.24)	0.008	1.78 (0.47-6.72)	NS
ErbB2 expression	Positive vs. negative	2.33 (1.30-4.18)	0.005	1.75 (0.87-3.53)	NS
pErbB3 expression	Positive vs. negative	2.46 (1.37-4.41)	0.003	1.12 (0.53-2.38)	NS
ErbB4 expression	Positive vs. negative	2.99 (1.67-5.37)	<0.001	1.42 (0.55-3.65)	NS
pErbB4 expression	Positive vs. negative	3.84 (2.11-6.99)	<0.001	2.59 (1.01-6.69)	0.049

## Discussion

We examined the expression, localization, and clinical significance of heregulin, EGFR, pEGFR, ErbB2, pErbB2, ErbB3, pErbB3, ErbB4 and pErbB4 in CRC. Although there have been a number of reports regarding the expression of ErbB family members in CRC, there have been few studies targeting the phosphorylated ErbB family members; furthermore, the number of cases examined in those studies was relatively small and the association with patient prognosis was not elucidated [28]. We examined the relationship between the expression of phosphorylated ErbB family members and clinicopathological characteristics of CRC patients using a relatively large number of cases. As a result, we have shown that EGFR, ErbB2, ErbB3 and ErbB4 expression in CRC patients was positive in 51%, 26%, 36% and 21% cases, respectively, consistent with previous reports. In addition, we found positive pEGFR, pErbB2, pErbB3 and pErbB4 expression in 32%, 21%, 18% and 16% of CRC patients, respectively. Notably, pEGFR, pErB2 and pErbB3 localized in the nuclei of CRC cells; and we found for the first time that pEGFR, pErbB2, pErbB3 and pErbB4 correlated with poorer patient prognosis.

We found that heregulin is expressed exclusively in colorectal cancer cells. This is inconsistent with a recent paper, in which heregulin was expressed exclusively in mesenchymal cells of CRC tissues [24]. Although we did not exclude the possibility of heregulin expression in mesenchymal cells, we believe that heregulin originating from cancer cells mainly contribute to tumorigenesis. Previous studies have reported that interleukin-1, prostaglandin E_2_, hepatocyte growth factor and keratinocyte growth factor can upregulate the expression and secretion of heregulin in epithelial cells [29]. We have already examined heregulin expression using a number of cancer cell lines and biopsy specimens of colorectal cancer tissues and found that cancer epithelial cells themselves express heregulin in situ [23]. In addition, we found that heregulin and vascular endothelial growth factor colocalized in cancer epithelial cells in CRC patients. Other investigators also have shown that heregulin is co-expressed with ErbB2 expression in colon cancer specimens and that autocrine activation of ErbB2 occurs through dimerization to ErbB3 in a colon carcinoma cell line [30]. This suggests that autocrine heregulin/ErbB family loops may be important modulators of aberrant growth in colon cancer.

Although EGFR is the only ErbB family member with a clinically validated role in CRC, there is a wide variation in the expression level (from 8 to 100%) of EGFR and there is no data regarding pEGFR localization by immunohistochemical analysis [4]. Also, there are conflicting data on its prognostic significance [31]. Here, we show that pEGFR but not EGFR expression correlated with worse patient overall survival rate both in univariate and multivariate analysis using the Cox proportional hazards model.

Several studies have reported the overexpression of ErbB2 in CRC and there is a wide variation in the expression level of ErbB2 [4, 32]. Furthermore, unlike breast cancer, heterogeneous ErbB2 immunostaining has been reported in CRC [33]. Conradi et al. concluded that ErbB2 status should ideally be analyzed on representative slides of the resected tumor and that biopsy samples may not be sufficient [32]. We used representative slides of surgically resected CRC samples to avoid sampling error due to small sample size.

ErbB2 is detected in the cytoplasmic fraction of Caco-2 cells in steady state. However, when Caco-2 cells are stimulated with exogenous heregulin, pErbB2 is detected in the nuclear fraction as well as in the cytoplasm fraction of these cells. In fact, immunohistochemical analysis has detected positive pErbB2 expression both in the cytoplasm and nucleus of CRC tissues. This suggests that ErbB2 localized in the cell membrane translocates partially to the nucleus via ligand stimulation. Therefore, we did not consider ErbB2 in the cytoplasm and nuclei of cancer cells as positive; rather, we determined pErbB2 in the cytoplasm and nuclei of these cells to be positive.

As a result, we found that pErbB2 but not ErbB2 significantly correlated with disease free survival of CRC patients. These results suggest that ErbB2 phosphorylated through dimerization to ErbB3 stimulated by heregulin accounts for the aggressive behavior of CRC cells.

ErbB3 was found by immunohistochemical analysis to be localized mainly in the cell membrane of CRC cells, consistent with a recent report [34]. On the other hand, pErbB3 was found exclusively in the nucleus of CRC cells. There have been few studies regarding the localization of pErbB3 in CRC [28]. Those studies reported that pErbB3 was observed in the nucleus of CRC cells in 22.7% cases, which is consistent with our data. We examined whether ErbB3 or pErbB3 expression is related to patient prognosis. Previous data is controversial; Baiocchi et al. have shown that ErbB3 expression is related to better overall survival, a finding that has not been confirmed by other studies [34–36]. Using ErbB3 knockdown mice, Lee et al. have shown that ErbB3 has essential roles in supporting intestinal tumorigenesis and suggest that ErbB3 may be a promising target for the treatment of colorectal cancers [9]. We show that pErbB3 but not ErbB3 expression correlated with worse patient disease free survival rate both in univariate and multivariate analysis using the Cox proportional hazards model.

There have been a few reports concerning ErbB4 in CRC tumorigenesis. There have been only four studies on ErbB4 expression patterns and prognostic significance in CRC patients [36–39]. Baiocchi et al. examined 109 stage II-III CRC patients for the expression levels of EGFR to ErbB4 and found membranous positive ErbB4 expression as an independent prognostic factor for recurrence, while other researchers did not find any significant relationship between ErbB4 expression and patients prognosis. There has been no report to date concerning pErbB4 expression patterns and prognostic significance in CRC patients. In the present study, we found for the first time that both ErbB4 and pErbB4 positivity is significantly related with worse prognosis. In particular, we show that pErbB4 expression correlated with worse patient disease overall survival rate in multivariate analysis using the Cox proportional hazards model independent of pEGFR expression or lymph node metastasis. This suggests that the heregulin-ErbB4 loop affects CRC patients’ prognosis to the same degree as that of EGFR activation.

It appears that ErbB4 protein expression is difficult to detect in cultured cancer cell lines although there have been a few reports that ErbB4 can be detected in certain CRC cell lines [27]. In fact, we also have reported that ErbB4 is almost negative in the cytosolic fractions of two CRC cell lines examined, DLD-1 and Caco-2 [23]. This corresponds with the fact that few CRC cell lines express ErbB4. In the present study, we chose HCT 116 according to a previous report [27]. In addition, we used concentrated protein lysate to detect ErbB4 protein more effectively. As a result, we have shown that both ErbB4 and pErbB4 stimulated by heregulin can be detected in the cytoplasm of HCT 116 cells.

Accumulating evidence suggests that EGFR translocates to the nucleus both in normal and cancer cells alike. Nuclear EGFR has been identified in various tumor tissues, including breast cancer, ovarian cancer, and oropharyngeal and esophageal squamous cell carcinomas, and has been shown to be associated with poor patient outcomes [40]. Here, we show, by immunoprecipitaion and western blot analysis, that ligands stimulate the translocation of ErbB2 and ErbB3 into the nucleus in CRC cells. The molecular weight of pErbB2 and pErbB3 in the nuclear fraction was approximately 180 kD, corresponding to the molecular weight of the full-length ErbB2 (185 kD) and ErbB3 (180 kD). The DLD-1 cells express EGFR, ErbB2, and ErbB3 strongly. On the other hand, The Caco-2 cells express ErbB2 and ErbB3 weakly, and EGFR negative. pErbB2 was observed without heregulin stimulation in the nuclear fractions of DLD-1 cells, whereas pErbB2 and pErbB3 were detected in the nuclear fractions of Caco-2 cells only after heregulin stimulation. These results suggest that heregulin stimulated ErbB2 and ErbB3 phosphorylation in the nuclear fractions without participation of the EGFR signal crosstalks since Caco-2 cells express low levels of EGFR [23]. These data suggest that, in addition to EGFR, ErbB2 or ErbB3 itself may play a role as a signal transduction mediator. Unlike EGFR and ErbB2, ErbB3 has been known as a kinase-inactive member of the EGFR family, which lacks the ability of signal transduction without ErbB2 or ErbB4. However, our immunohistochemical analysis has shown that ErbB3 does not always colocalize with ErbB2 or ErbB4. Thirty-one cases (20%) were positive for only ErbB3. Therefore, it is reasonable to consider that ErbB3 alone may play a role via its translocation to the nucleus of cancer cells, where it can target a particular gene to induce the expression of a specific oncoprotein.

## Conclusions

We have shown that phosphorylated ErbB2 and ErbB3 stimulated by heregulin, localized in the nucleus of CRC cells. Phosphorylated EGFR, ErbB2, ErbB3, ErbB4 and their relevant growth factor heregulin contribute to worse patient prognosis in CRC. This heregulin-ErbB family member autocrine loop may be a candidate target of CRC therapy as well as EGFR. Further investigation is clearly warranted to elucidate the role of nuclear phosphorylated ErbB2 and ErbB3 in CRC.

## Electronic supplementary material

Additional file 1: Table S1: List of primary antibodies used in this study. (DOCX 241 KB)

Additional file 2: Table S2: Relationship between ErbB1-4, phosphorylated ErbB1-4 and heregulin. (DOCX 162 KB)

## Abbreviations

EGFR: Epidermal growth factor
CRC: Colorectal cancer
pErbB: Phosphorylated ErbB
rHRG: Recombinant human heregulin-β1 EGF domain
IgG: Immunoglobulin.
