# Supplementary material for: Localization of phosphorylated ErbB1-4 and heregulin in colorectal cancer
Source: BMC Cancer. 2014 Nov 22;14:863. doi: 10.1186/1471-2407-14-863 (PMC4247672; doi:10.1186/1471-2407-14-863)
Supplement: Supplementary file 1 — Additional file 1: Table S1: List of primary antibodies used in this study. (DOCX 241 KB) [file 12885_2014_5035_MOESM1_ESM.docx]

Additional file 1/Table S1 List of primary antibodies used in this study
