# Supplementary material for: Localization of phosphorylated ErbB1-4 and heregulin in colorectal cancer
Source: BMC Cancer. 2014 Nov 22;14:863. doi: 10.1186/1471-2407-14-863 (PMC4247672; doi:10.1186/1471-2407-14-863)
Supplement: Supplementary file 2 — Additional file 2: Table S2: Relationship between ErbB1-4, phosphorylated ErbB1-4 and heregulin. (DOCX 162 KB) [file 12885_2014_5035_MOESM2_ESM.docx]

Additional file 2/Table S2 Relationship between ErbB1-4, phosphorylated ErbB1-4 and heregulin
